# Temporal Heterogeneity of Short-Term Effects of Particulate Matter on Stroke Outpatients in Seven Major Cities of the Republic of Korea

**DOI:** 10.3390/ijerph191912316

**Published:** 2022-09-28

**Authors:** Yongsoo Choi, Garam Byun, Jong-Tae Lee

**Affiliations:** 1 School of Health Policy and Management, College of Health Science, Korea University, 145, Anam-ro, Seongbuk-gu, Seoul 02481, Korea; 2 Interdisciplinary Program in Precision Public Health, Korea University, 145, Anam-ro, Seongbuk-gu, Seoul 02481, Korea

**Keywords:** air pollution, temporal variation, cerebrovascular disease, stroke, health effects

## Abstract

Although particulate matter (PM) is a major risk factor for stroke, its effects on hospital outpatients admitted for stroke have not been documented in Korea. In addition, recent studies have reported that the effects of PM_10_ on circulatory mortality changed over time. We aimed to estimate the effects of PM_10_ on stroke and their temporal heterogeneity in seven major cities of Korea during the period 2002–2015. The study period was divided into five years of moving time windows, and city-specific PM_10_ effects on ischemic and hemorrhagic stroke outpatients were calculated. We pooled the estimates using meta-analysis and plotted them into a sequence to identify their temporal trends. A 10 µg/m^3^ increase of PM_10_ was significantly associated with increments in hospital outpatients admitted for ischemic stroke (0.24%, 95% CI: 0.04%, 0.44%), but not for hemorrhagic stroke (0.33%, 95% CI: −0.06%, 0.73%). Effect estimates for strokes increased during the period 2003–2013 but decreased after. For the first time, we have estimated the effects of PM_10_ on hospital outpatients admitted for stroke in Korea. The observed temporal trend in PM_10_ effects was similar to patterns of circulatory mortality, suggesting that the temporal heterogeneity in PM_10_ effects might be due to systematic causes rather than random fluctuations.

## 1. Introduction

Stroke is one of the top leading causes of death globally [1]. It is the second-highest cause of death in Korea and the second-largest public health burden in terms of the disability-adjusted life year (DALY) [2].

There are several risk factors for stroke. Poor diet, high blood pressure, smoking, and ambient particulate matter (PM) would be major risk factors for stroke [3]. Among them, the importance of PM is becoming higher in Korea. Not only does PM have adverse health effects on various health outcomes, but also Korea has experienced a severe level of PM exposure compared to other metropolitan cities [4,5]. According to the Organization for Economic Cooperation and Development (OECD), the pollution level in Seoul was approximately two times higher than that of other major cities in developed countries [6]. Even though the individual risk of PM is small, the overall population attributable risk and the subsequent burden are not ignorable because they are unavoidable, and the exposure lasts for a lifetime [3].

Studies of systemic reviews and meta-analysis covering various countries have reported significant associations between short-term exposure to PM and stroke [7,8]. However, the harmful effect of PM on stroke has not been well documented in Korea. To our best knowledge, there have been three published works regarding the effects of PM on stroke mortality in Korea. However, they had limitations: they were based on a single city (Seoul) and were from 20 years ago [9,10,11]. For outpatients, we could not find any published work regarding the effects of PM on stroke.

Furthermore, recent studies from several countries have suggested that PM_10_ effects on mortality might have changed over time [12,13,14,15,16,17,18,19,20]. Departing from the change of mass concentration, they suggested that the relative risk by a unit mass increase of PM_10_ can change over time. If temporal heterogeneity of the PM_10_ effect exists, estimating risks using all the available data (e.g., over a decade) without considering the heterogeneity would result in an average risk that fails to represent both the current and the upcoming risk. In addition, effect estimates calculated from old studies might not adequately represent the present risk of PM_10_.

Thus, in this study, we aimed to estimate the effects of PM_10_ on hospital outpatients admitted for stroke and investigate whether the relative risk of PM_10_ changed over time, from 2002 to 2015, in seven major cities in Korea.

## 2. Materials and Methods

### 2.1. Study Population

The study area consisted of the seven major cities of Korea, including Seoul, Busan, Daegu, Incheon, Gwangju, Daejeon, and Ulsan. The cities are representative urban cities of Korea that have measured PM concentrations for over a decade. They cover 46% of the Korean population (22,378,352 people) [21]. The population of each city is presented in Table 1.

### 2.2. Exposure: PM_10_

Hourly data of ambient PM_10_ concentrations, measured by the β-ray absorption method, were obtained from the National Institute of Environmental Research (NIER). Because NIER has several measurement stations, city-specific hourly PM_10_ concentrations were calculated by first averaging the hourly data within each city. Then, daily 24-h mean concentrations were calculated by averaging the previously calculated hourly values. Hourly temperature and relative humidity data, measured by the automated surface observing system (ASOS), were obtained from the Korea Meteorological Administration (KMA). KMA has one representative ASOS in each of the seven major cities. The daily 24-h mean temperature and relative humidity were calculated.

### 2.3. Outcomes: Stroke Outpatients

The number of daily hospital outpatients admitted for stroke was obtained using health insurance claim data from the National Health Insurance Service (NHIS). One of the strengths of NHIS data is that it covers almost the entire Korean population (99.7% in 2006) [22]. Diseases were classified using the principal diagnostic code of the International Classification of Disease (ICD-10), as follows: I61 (hemorrhagic stroke) and I63 (ischemic stroke). We excluded those aged over 100 years and whose hospital address differed from the residential address. Medical records were used only from the hospital, clinic, and health center. Records from the general hospital were excluded because outpatients for general hospitals in Korea are most likely to be planned visits rather than be due to abrupt symptoms. When a record has the same diagnosis as the previous one within 31 days for a person, we excluded the latter because it is likely to be a follow-up rather than an incidence case [23].

### 2.4. Statistical Analysis

Statistical analyses were carried out in three different stages. In the first stage, city-specific relative risks were estimated using the autoregressive time-series model assuming quasipoisson distribution for 2002–2015 [24,25]. Days with the Asian dust intrusion were excluded, as previous research indicated it could underestimate the effects of PM_10_ [26,27]. We trimmed the upper and lowered 0.5% of extremes when the residuals were not normally distributed. The detailed statistical model is as follows:

(1) $$g\left[E\left(Y_{i,j}\right)\right]=\beta_{0}+\beta_{1}PM_{i,j}+ns\left(Time_{i},\;df=6/year\right)+ns\left(Temp_{i},\;df=4\right)+\beta_{2}RH_{i,j}+D_{1}DOW_{i}+D_{2}Holidays_{i}+\beta_{3}Residual_{i,j}$$

where *g*[.] is a generalized linear function with a quasipoisson link and $Y_{i,j}$ denotes the number of outpatient visits on day i and city j. We adjusted for long-term time trend (Time) and temperature (Temp) using a natural cubic spline (ns) with fixed degrees of freedom (df). Relative humidity (RH) was adjusted using a linear term, and the day of the week (DOW) and national holidays (Holidays) were adjusted using dummy variables. We further adjusted lagged deviance residuals (Residual) to address the residual autocorrelation problem.

In the second stage, the city-specific relative risks were summarized using a random effect meta-analysis to estimate. We tested the temporal heterogeneity of PM_10_ risk in the third stage. The study period was divided into five years of moving time windows (2002–2006, 2003–2007, …, 2011–2015), and then the previous two-stage analysis was re-conducted within each time window [14,16]. Pooled relative risks and the 95% confidence interval (95% CI) from each time window were plotted in sequence to delineate the temporal trend of the PM_10_ effects in the seven major cities of Korea [14,16]. A significant test between time windows was performed using a ratio of relative risks, assuming a normal distribution [28].

The estimated relative risks are presented as the percent change in risk ((relative risk-1) × 100) associated with a 10 µg/m^3^ increase in PM_10_, if not specified. All analyses were performed using the R software version 3.3.1 using “mgcv” (ver. 1.8-12) and “metafor” (ver. 2.0-0) packages [29].

Several sensitivity analyses were performed. First, nitrogen dioxide and sulfur dioxide were adjusted to consider potential confounding by other air pollutants. Second, lagged exposure of PM_10_ for up to 5 days was evaluated. The effects of cumulative lag (lag01, lag02, …, lag05), calculated by the moving average method, were tested. Lastly, as a lagged effect of temperature may confound the association of PM_10_ and health outcomes, we adjusted temperature exposure up to 21 days before using the distributed lag model (DLM) [30]. The temperature–response association was modelled using quadratic B-spline with internal knots placed at the 25th, 50th, and 90th percentile of temperature, and lag-response associations were modelled using cubic B-spline with the logarithm based-knots defined by 7 degrees of freedom [30,31].

## 3. Results

Table 2 shows the summary statistics of the study variables. The daily mean counts of hospital outpatient for ischemic stroke and hemorrhagic stroke were 30.9 and 2.5 in the seven major cities. For a temporal change, the number of ischemic stroke outpatient visits increased from 2002 to 2010 and remained stable. Outpatient visits for hemorrhagic stroke showed a similar temporal trend (Figure S1).

**Figure 1:**
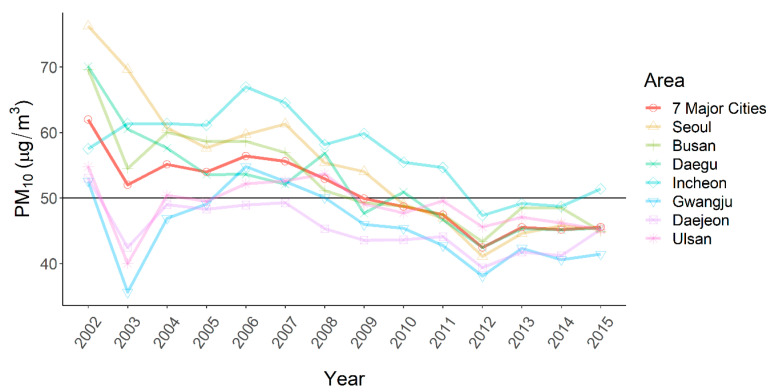
Annual averages of PM_10_ concentration in the seven major cities of Korea (2002–2015).

The daily average PM_10_ concentration in the seven major cities from 2002–2015 was 50.9 µg/m^3^. The annual average concentration of PM_10_ was 62.0 µg/m^3^ in 2002 and decreased to 45.5 µg/m^3^ in 2015. The decreasing temporal trend of PM_10_ concentration was similar over the seven major cities (Figure 1).

**Figure 2:**
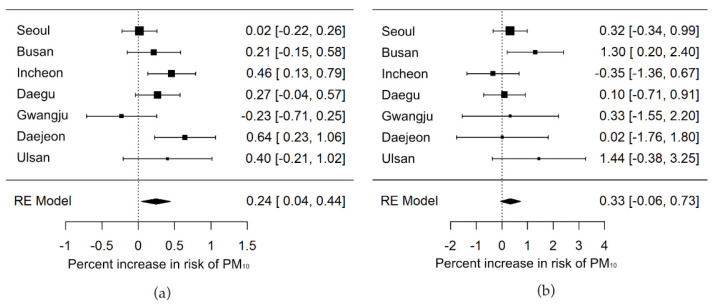
City-specific and pooled effect estimates of PM_10_ for stroke outpatients in the seven major cities of Korea, 2002–2015. (**a**) Hospital outpatients for ischemic stroke; (**b**) hospital outpatients for hemorrhagic stroke; RE model: Random effect model.

Figure 2 shows city-specific and pooled effect estimates for the short-term effects of PM_10_ on stroke. The 10 µg/m^3^ increase of PM_10_ was associated with a 0.24% (95% CI: 0.04%, 0.44%) increase in hospital outpatients for ischemic stroke for the period 2002–2015. However, we could not find statistically significant associations for hemorrhagic stroke (0.33%, 95% CI: −0.06, 0.72) (Figure 2). The effect estimates tend to attenuate when we adjust the other air pollutants (Tables S1 and S2). Adjusting temperature extensively using the DLM did not change the estimated results (Tables S1 and S2). A partly negative association between PM_10_ and stroke outpatient was observed in the lagged exposure, presenting a possibility for morbidity displacement (Figure S2).

**Figure 3:**
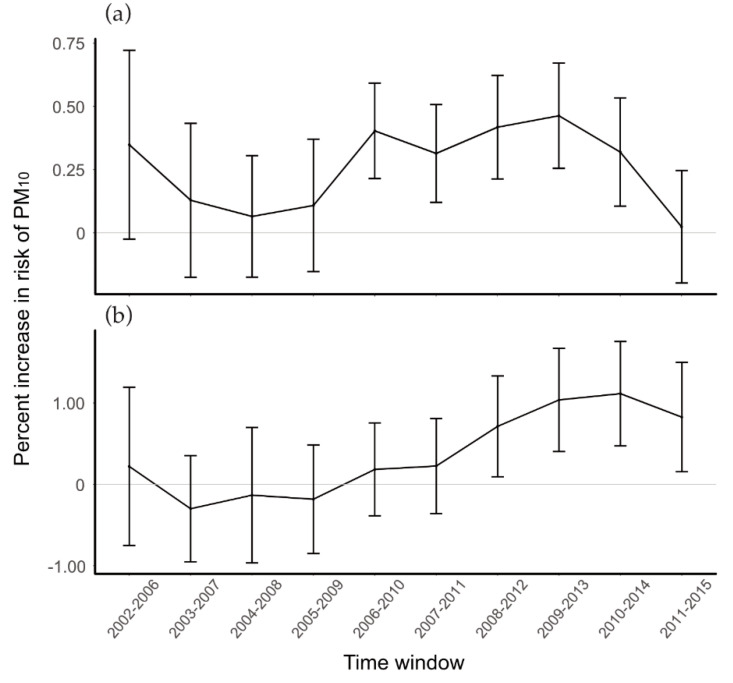
Temporal trend of PM_10_ effects on hospital outpatients due to stroke in seven major cities of Korea, 2002–2015. (**a**) Hospital outpatients admitted for ischemic stroke; (**b**) hospital outpatients admitted for hemorrhagic stroke.

Figure 3 shows the temporal trends of PM_10_ effects on hospital outpatients admitted for stroke. For ischemic stroke, effect size tends to increase for the 2004–2008 period (point estimate: 0.06%, 95% CI: −0.20%, 0.31%) until the 2009–2013 period (point estimate: 0.50%, 95% CI: 0.29%, 0.71%). The difference in effect estimates between the two periods was statistically significant (*p*-value < 0.01). Afterwards, however, the effect size decreased, and the association was no longer statistically significant in the 2011–2015 period (point estimate: 0.08%, 95% CI: −0.14%, 0.30%). The difference in the effect estimates from 2009–2013 to 2011–2015 was also statistically significant (*p*-value <0.01). For hemorrhagic stroke, the effect estimates showed an increasing temporal trend until the last time window. While the effect estimate of PM_10_ on hemorrhagic stroke for the entire study period was not statistically significant, the effect estimates from the most recent five years showed statistically significant associations. When we considered more prolonged lag exposure, the observed temporal trend did not change, while the effect sizes were attenuated (Figure S3).

## 4. Discussion

In the present study, we found positive relationships between short-term exposure to PM_10_ and daily hospital outpatients admitted for stroke in the seven major cities of Korea. In addition, we found evidence that the effect size of PM_10_ varied over time. It increased early in the study period and has decreased in recent years.

PM_10_ was significantly associated with ischemic stroke during the period 2002–2015, while hemorrhagic stroke was not. Compared to ischemic stroke, the epidemiological evidence linking hemorrhagic stroke and air pollution are relatively poor and inconsistent [7]. The underlying mechanism of PM for cerebrovascular diseases has not been fully elucidated and might differ for ischemic and hemorrhagic strokes. The classical “inflammation” hypothesis is that macrophages ingest particles inhaled through the lungs and activate inflammatory responses. Inflammation can impact the initiation, propagation, and inhibitory phases of blood coagulation and consequently increase the risk of ischemic stroke [32,33,34]. Other potential mechanisms linking air pollution to stroke include endothelial or autonomic dysfunction and nanoparticles, which can pass through alveolar-capillary barriers and affect the vasculature and circulating blood cells [7,32,35]. Although the association between PM_10_ and hemorrhagic stroke for the entire study period was not statistically significant, the association in recent years showed significant associations. The relationship between PM and hemorrhagic stroke is less grounded than ischemic stroke, but there are still some pieces of evidence on hemorrhagic stroke. A study suggests positive associations between PM_2.5_ and hemorrhagic stroke [36], and another study reported significant results between PM_2.5_ and hemorrhagic stroke during warm days (>13.5 °C) [37]. There is a possibility that the changes in the composition of PM or changes in the surrounding environment could lead to an increased risk of PM_10_ on hemorrhagic stroke.

Recently, an increasing number of studies from various countries have investigated temporal variability in the effect of PM. However, studies of temporal variation showed conflicting results by country. In some countries, researchers found an increasing temporal trend in PM effects [12,13,15,17], while others found a decreased temporal trend [19,20]. There was also a study that did not find signs of temporal change in PM effects [18].

In the case of Seoul, Korea, the effect of PM_10_ on circulatory mortality tended to increase until 2012, and decreased after [14]. The pattern derived from the mortality outcomes is similar to our results for stroke outpatients within an extended study area. Combining the evidence, the effects of PM_10_ in circulatory disease seem to have changed over time, and systematic factors rather than random fluctuations likely cause the change.

The temporal heterogeneity in risk is a phenomenon caused by various factors. Changes in population characteristics, PM characteristics, health policies, other pollutants, measurement error, and non-linear exposure–response relationships might all be related to heterogeneity in PM effects. Of these factors, changes in size and chemical composition of PM are suspected to be a relevant cause of the observed temporal variation in Korea. Choi et al. [14] compared the temporal trend of PM_2.5_ to PM_10_ ratio with PM_10_ mortality risks and found similarities. The different degrees of toxicity between PM_2.5_ and PM_2.5–10_, and the change in their ratio over time could alter the effects of PM_10_.

Besides the size distribution, changes in the chemical composition of PM could also cause heterogeneity. Unfortunately, it was not long ago that PM_2.5_ and its specific compositions were first measured in major cities other than Seoul. The absence of detailed data on the chemical composition of PM is one of the biggest obstacles in investigating the causes of temporal change in Korea. Nonetheless, a study found that city characteristics such as SO_2_, SO_2_/PM_10_, and GRDP partially explain the spatial heterogeneity of short-term effects of PM_10_ in Korea. They suggested that these characteristics may surrogate the toxicity of PM_10_ [38]. Future efforts using those proxy indicators to explain the temporal heterogeneity in PM_10_ effects might help overcome the limitation of data absence for the PM compositions.

The causes of risk heterogeneity should be explored and interpreted under the socio-ecological and environmental context of the study area. For the last five years of the study period, our effect estimate of PM_10_ was not significantly associated with cerebrovascular outpatients. However, this might not necessarily mean that PM_10_ is no longer harmful to our health. Instead, the last decrease in PM_10_ risk might be attributable to an increased exposure measurement error. After the WHO announced outdoor air pollutants as a Group 1 carcinogen in 2013, public awareness regarding PM increased significantly in Korea [39]. In addition, the Ministry of Environment implemented a nationwide particulate matter alert system in 2014. Based on the alerts, people might have begun to take personal precautionary measures, which would increase the gap between ambient PM_10_ concentrations and personal exposures. Further studies using various exposure measurement methods would be essential to elaborate on recent PM risks in Korea.

Our findings on the temporal variation of PM effects have several public health implications. When the temporal variation of PM risk exists, estimating one overall risk value for the entire study period would result in an average risk that does not represent any actual risks. In these circumstances, it would be desirable to contemplate the causes of temporal variation and then estimate the risks by dividing the study period into homogeneous risk periods.

In addition, it can be seen that exposure management is not the only answer in the PM management strategy. Even if the ambient concentration decreases, the expected health benefits may not be achieved if the relative risk of PM increases. To minimize the adverse health effects caused by PM, public health policies should contemplate ways to minimize both exposure and relative risk.

Finally, a quantitative risk assessment of an environmental risk factor is an essential scientific basis for public health policies. When using research-derived quantitative evidence in a policy cycle, it should be considered not only how valid the causal estimates are in a study population (i.e., internal validity) but also whether the measured causal effect would remain the same in other populations, places, or times (i.e., external validity) [40]. Questions about external validity are often raised when a result from a study is applied to another country or region. However, it is rarely questioned when applying a study result at a particular period to another time. Temporal heterogeneity in risk should also be considered when applying epidemiologic evidence to policy cycles.

There are several strengths and limitations relevant to this study. The present study is the first to investigate the effects of PM_10_ on hospital outpatients for stroke in Korea. In addition, we used national insurance claim data that covers almost the entire Korean population. Regarding the limitations, it was impossible to distinguish whether the hospital records were incidence cases or planned visits for follow-up symptoms. The inability to use emergency room data is also a limitation. To avoid bias from the planned visit, we restricted the types of hospitals and applied the 31-day exclusion criteria. Analysis for PM_2.5_ was not possible due to the lack of data.

## 5. Conclusions

The present study showed that short-term exposure to ambient PM_10_ is positively associated with hospital outpatients admitted for stroke, and the effect size might have changed over time in Korea. Given that the observed temporal trends in PM_10_ effects are similar to those of previous studies using mortality outcomes in Korea, the discovered temporal trend is likely to be caused by systematic factors rather than random fluctuations. To accurately estimate the current or upcoming risk of PM, further studies should explore the causes of the temporal variation from a context-based perspective.

## Figures and Tables

**Table 1:** Descriptive information for the seven major cities of Korea in 2010.

City	Population (Count)	Area (km^2^)	Number of Air Pollution Monitoring Stations
Seoul	9,794,304	605	25
Busan	3,414,950	767	17
Daegu	2,446,418	884	11
Incheon	2,662,509	1029	15
Gwangju	1,475,745	501	7
Daejeon	1,501,859	540	7
Ulsan	1,082,567	1059	13

**Table 2:** Daily averages of the study variables and their standard deviations during the 2002–2016.

	Ischemic Stroke Outpatients	Hemorrhagic Stroke Outpatients	PM_10_ (µg/m^3^)	Temperature (°C)	Relative Humidity (%)
Mean	30.9 (30.4)	2.5 (3.2)	50.9 (33.2)	13.8 (9.4)	63.9 (16.3)
Seoul	56.2 (36.9)	4.3 (3.7)	54.8 (41.2)	12.8 (10.4)	61.0 (14.9)
Busan	36.3 (23.5)	2.7 (2.5)	52.9 (31.0)	14.9 (8.1)	62.0 (18.4)
Incheon	23.5 (16.6)	2.2 (2.4)	57.0 (33.6)	12.6 (9.8)	69.5 (15.5)
Daegu	58.1 (41.1)	5.6 (4.5)	52.0 (31.7)	14.6 (9.5)	57.6 (16.7)
Gwangju	14.6 (9.6)	0.9 (1.1)	45.6 (30.0)	14.2 (9.4)	67.2 (13.2)
Daejeon	17.2 (10.7)	0.8 (1.1)	45.4 (30.5)	13.1 (9.9)	67.0 (14.2)
Ulsan	10.7 (8.5)	0.8 (1.1)	48.8 (30.8)	14.5 (8.7)	62.7 (17.4)

## Data Availability

Data in this study were from the National Health Insurance Service, National Institute of Environmental Research, and Korea Meteorological Administration.

## Supplementary Materials

The following supporting information can be downloaded at: https://www.mdpi.com/article/10.3390/ijerph191912316/s1, Figure S1: Temporal trend of hospital outpatient visits for strokes, seven major cities of Korea, 2002–2015; Figure S2: Lag effects of PM10 on hospital outpatients due to strokes in the seven major cities of Korea, 2002–2015. By different lag effects; Figure S3: Temporal trend of PM10 effects on hospital outpatients due to strokes in the seven major cities of Korea, 2002–2015. By different lag effects; Table S1: Percent increase of PM10 for hospital outpatients due to ischemic stroke, the seven major cities of Korea, 2002–2015; Table S2: Percent increase of PM10 for hospital outpatients due to hemorrhagic stroke, the seven major cities of Korea, 2002–2015.
