# Supplementary material for: Temporal Heterogeneity of Short-Term Effects of Particulate Matter on Stroke Outpatients in Seven Major Cities of the Republic of Korea
Source: Int J Environ Res Public Health. 2022 Sep 28;19(19):12316. doi: 10.3390/ijerph191912316 (PMC9566257; doi:10.3390/ijerph191912316)
Supplement: Supplementary file 1 [file ijerph-19-12316-s001.zip › ijerph-1920805-supplementary.pdf]

ONLINE SUPPLEMENT

Article

Temporal heterogeneity of Short-Term Effects of the Particulate Matter on Stroke outpatients in Seven Major Cities of the Republic of Korea

Yongsoo Choi [1], Garam Byun [2] and Jong-Tae Lee [1,2,*]

[1] School of Health Policy and Management, College of Health Science, Korea University, 145, Anam-ro, Seongbuk-gu, Seoul 02481, Korea
[2] Interdisciplinary Program in Precision Public Health, Korea University, Seoul 02481, Korea
* Correspondence: jtlee@korea.ac.kr; Tel.: +82 2 940 2770

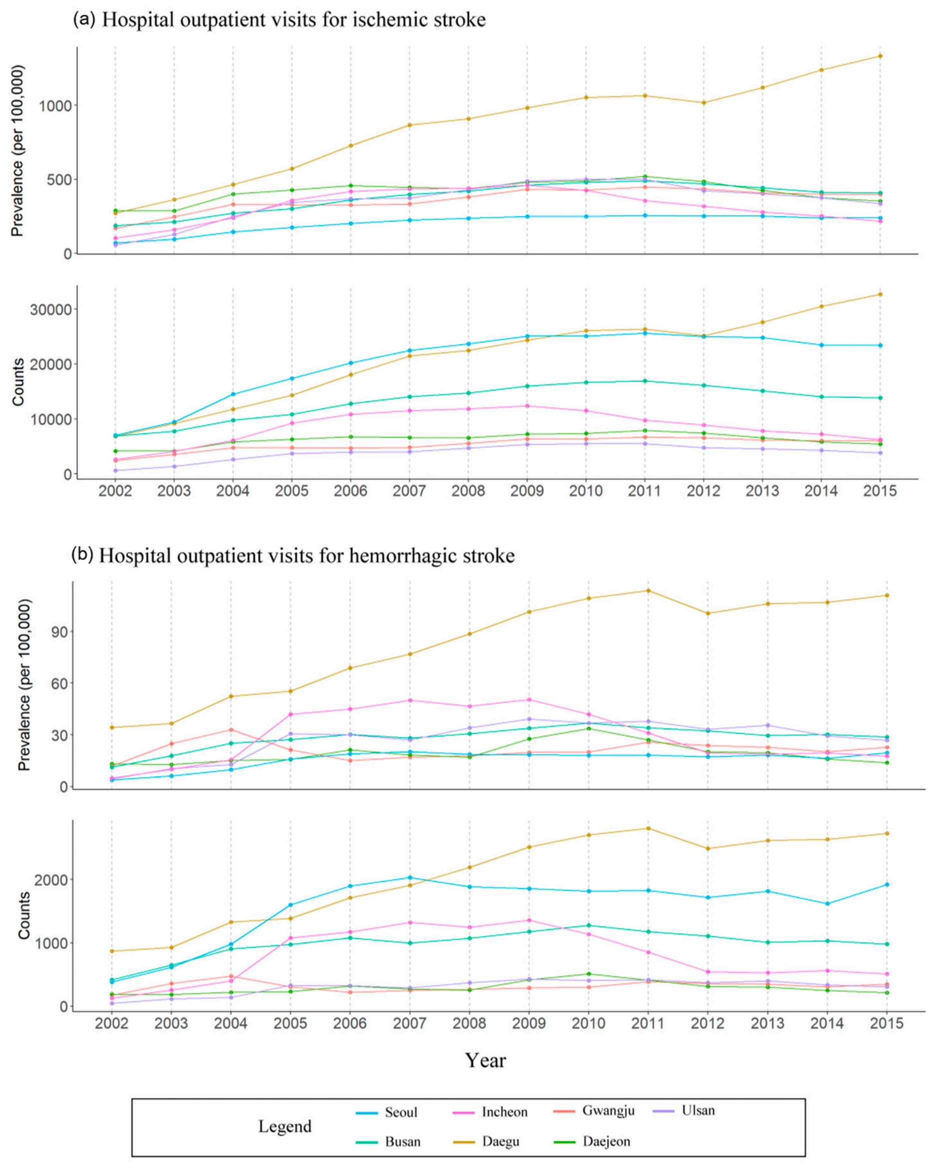

Figure S1. Temporal trend of hospital outpatient visits for the strokes, seven major cities of Korea, 2002-2015.

Table S1. Percent increase of PM_{10} for hospital outpatients due to ischemic stroke, the seven major cities of Korea, 2002-2015.

Area	PM_{10}	PM_{10} + NO_2 [a]	PM_{10} + SO_2 [b]	Temp (DLM) [c]
Pooled	0.24 (0.04, 0.44)	0.18 (0.01, 0.35)	0.20 (-0.04, 0.45)	0.22 (0.02, 0.41)
Seoul	0.02 (-0.22, 0.26)	-0.03 (-0.32, 0.25)	-0.08 (-0.41, 0.25)	-0.07 (-0.32, 0.18)
Busan	0.21 (-0.15, 0.58)	0.37 (-0.06, 0.79)	-0.04 (-0.50, 0.43)	0.34 (-0.03, 0.71)
Incheon	0.46 (0.13, 0.79)	0.42 (0.03, 0.80)	0.42 (-0.04, 0.87)	0.41 (0.08, 0.75)
Daegu	0.27 (-0.04, 0.57)	0.11 (-0.20, 0.43)	0.18 (-0.22, 0.58)	0.20 (-0.11, 0.51)
Gwangju	-0.23 (-0.71, 0.25)	-0.15 (-0.70, 0.40)	-0.10 (-0.68, 0.48)	-0.22 (-0.71, 0.27)
Daejeon	0.64 (0.23, 1.06)	0.60 (0.08, 1.12)	0.99 (0.43, 1.56)	0.67 (0.21, 1.13)
Ulsan	0.40 (-0.21, 1.02)	0.05 (-0.72, 0.83)	0.23 (-0.49, 0.96)	0.31 (-0.30, 0.92)

a. Two-pollutant model adjusting nitrogen dioxide
b. Two-pollutant model adjusting sulfur dioxide
c. Adjusting lag exposure of temperature up to 21 days

Table S2. Percent increase of PM_{10} for hospital outpatients due to hemorrhagic stroke, the seven major cities of Korea, 2002-2015.

Area	PM_{10}	PM_{10} + NO_2 [a]	PM_{10} + SO_2 [b]	Temp (DLM) [c]
Pooled	0.33 (-0.06, 0.73)	0.31 (-0.13, 0.76)	0.56 (-0.32, 1.44)	0.25 (-0.15, 0.65)
Seoul	0.32 (-0.34, 0.99)	0.23 (-0.56, 1.03)	0.23 (-0.69, 1.15)	0.21 (-0.48, 0.89)
Busan	1.30 (0.20, 2.40)	1.47 (0.19, 2.76)	2.20 (0.82, 3.58)	1.34 (0.22, 2.45)
Incheon	-0.35 (-1.36, 0.67)	0.14 (-1.04, 1.32)	-0.71 (-2.13, 0.71)	-0.41 (-1.45, 0.63)
Daegu	0.10 (-0.71, 0.91)	-0.16 (-0.98, 0.67)	-0.17 (-1.23, 0.89)	-0.04 (-0.88, 0.79)
Gwangju	0.33 (-1.55, 2.20)	0.28 (-1.87, 2.43)	1.92 (-0.32, 4.16)	0.06 (-1.85, 1.97)
Daejeon	0.02 (-1.76, 1.80)	0.11 (-1.96, 2.18)	-1.08 (-3.38, 1.20)	0.24 (-1.59, 2.06)
Ulsan	1.44 (-0.38, 3.25)	1.93 (-0.41, 4.26)	2.23 (0.05, 4.40)	1.27 (-0.58, 3.11)

a. Two-pollutant model adjusting nitrogen dioxide
b. Two-pollutant model adjusting sulfur dioxide
c. Adjusting lag exposure of temperature up to 21 days

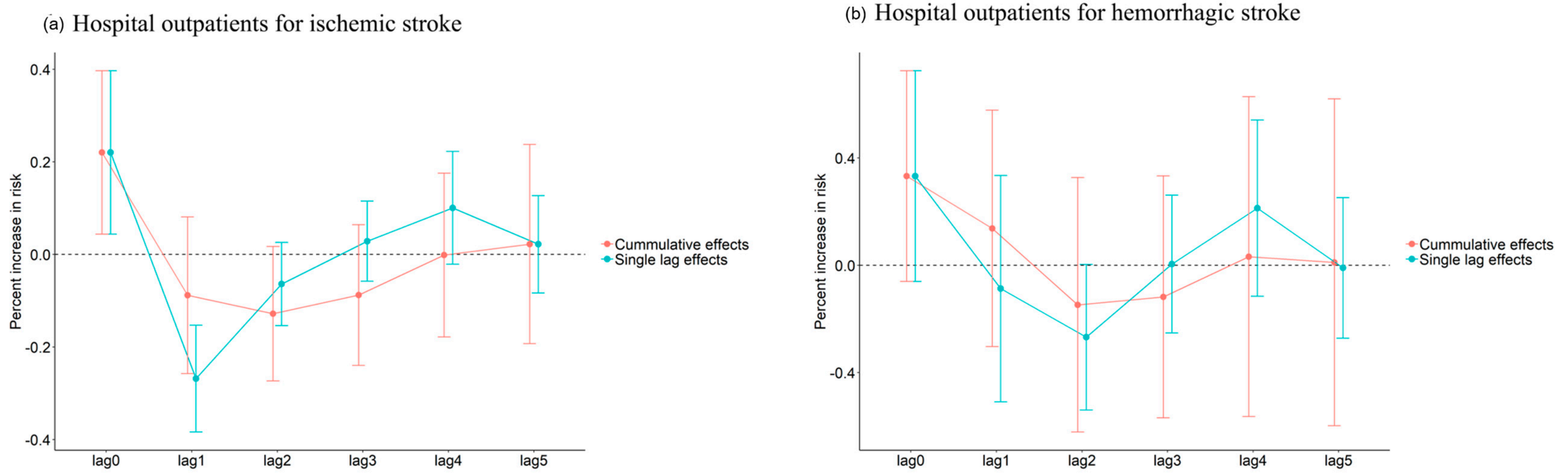

Figure S2. Lag effects of PM_{10} on hospital outpatients due to strokes in the seven major cities of Korea, 2002-2015. By different lag effects.

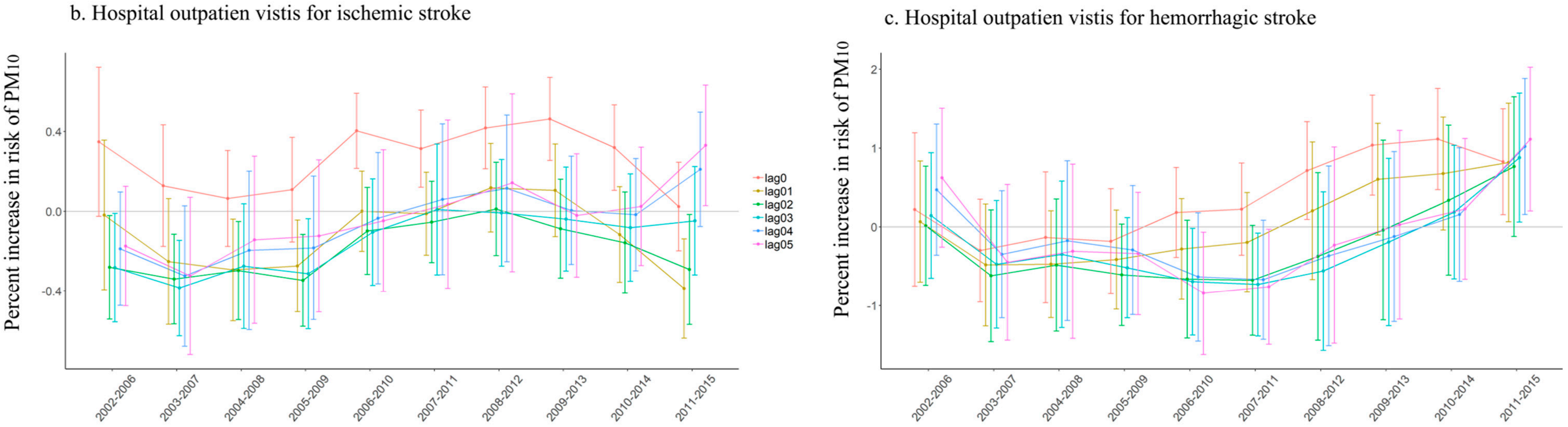

Figure S3. Temporal trend of PM_{10} effects on hospital outpatients due to strokes in the seven major cities of Korea, 2002-2015. By different lag effects.
